# Competence-Based Assessment of Biomedical Equipment Management and Maintenance System (e-Upkaran) Using Benefit Evaluation Framework

**DOI:** 10.7759/cureus.30579

**Published:** 2022-10-22

**Authors:** Pankaj Bhardwaj, Nitin K Joshi, Prem Singh, Praveen Suthar, Vibha Joshi, Yogesh K Jain, Jaykaran Charan, Mohammad Ameel, Kuldeep Singh, Manoj S Patil, Abhay Gaidhane, Zahiruddin Quazi Syed, Deepak Saxena

**Affiliations:** 1 Department of Community Medicine and Family Medicine, School of Public Health, All India Institute of Medical Sciences, Jodhpur, IND; 2 Department of Public Health, National Health Mission, Jodhpur, IND; 3 School of Public Health, All India Institute of Medical Sciences, Jodhpur, IND; 4 Resource Centre Health Technology Assessment, All India Institute of Medical Sciences, Jodhpur, IND; 5 Department of Pharmacology, All India Institute of Medical Sciences, Jodhpur, IND; 6 Department of Public Health, PATH (Program for Appropriate Technology in Health), New Delhi, IND; 7 Department of Pediatrics, All India Institute of Medical Sciences, Jodhpur, IND; 8 Department of Research and Development, Jawaharlal Nehru Medical College, Datta Meghe Institute of Medical Sciences, Wardha, IND; 9 School of Epidemiology and Public Health, Jawaharlal Nehru Medical College, Datta Meghe Institute of Medical Sciences, Wardha, IND; 10 Department of Community Medicine, Jawaharlal Nehru Medical College, Datta Meghe Institute of Medical Sciences, Wardha, IND; 11 Department of Public Health, Indian Institute of Public Health Gandhinagar, Gandhinagar, IND

**Keywords:** maintenance system, equipment management, biomedical equipment, rajasthan, management, biomedical, digital, efficiency, e-upkaran

## Abstract

Introduction

To establish a centralized inventory management system for the efficient functioning of all healthcare facilities, e-Upkaran (equipment management and maintenance system) was launched in 2015 in the state of Rajasthan. This study is conducted to assess the functioning of e-Upkaran in Rajasthan.

Methods

The assessment of the e-Upkaran system for primary and secondary healthcare centers was carried out using a systematic review of the literature and a multi-indicator stakeholder questionnaire. The benefits evaluation framework focused on the system quality, information and service quality, use and user satisfaction, and net benefits utilized for the assessment. A review of the literature was done to highlight the importance of computerized medical equipment management and maintenance systems and appraise the challenges and benefits associated with such systems as compared to the traditional pen-paper register. Information was gathered based on available documents, field observation, and data obtained from specific hospital staff, including the bioengineers and other users of e-Upkaran.

Results

The finding of this study suggests that e-Upkaran efficiently improves documentation, reporting, maintenance, and management of medical equipment. It is more efficient than the traditional paper-pen system. It is designed to minimize downtime and maintain equipment in good operating condition and has potential benefits in terms of improving information quality, use, and net benefit. The cost of service ratio is within the benchmark value. This system has also considerably reduced out-of-pocket expenditure. Computer proficiency and the workload of other e-health programs pose a challenge in the implementation of this program.

Conclusion

The e-Upkaran system is competent in terms of improving information quality, use, and net benefit. Other Indian states could also adopt this system to improve their biomedical equipment management and maintenance system.

## Introduction

According to the World Health Organization (WHO), the provision of equipment and diagnostics at a healthcare facility is as essential as the provision of trained medical professionals for an efficient health system [1]. The Government of Rajasthan has made provisions for free drugs and free diagnostic investigations under various schemes, but the beneficiaries are not able to avail of the services either due to absence or due to non-functional investigative machinery [2,3]. It was observed that at some facilities, the devices have been non-functional for a long time, and despite multiple requests for repair and maintenance, the department has shown a cold shoulder every time [3]. Hence, there was an urgent need to establish a centralized inventory management system for the efficient functioning of all healthcare facilities.

Therefore, e-Upkaran: Equipment Management and Maintenance System (EMMS), a complete system for equipment management and maintenance, was developed. e-Upkaran was launched on 2nd October 2015 by the Hon’ble Health Minister [4]. EMMS is an end-to-end web-based solution with a life cycle approach to equipment. It is an online platform for monitoring goods and services contracts, maintaining process transparency for generating bills, and setting accountabilities of various stakeholders involved in equipment procurement and maintenance [4]. Since it has been launched, limited efforts have been made to assess the functioning of the e-Upkaran centralized inventory management system. Given this, the present study is conducted to assess the functioning of e-Upkaran, which will allow us to gain insight into how competently the system is performing.

## Materials and methods

In this study, an assessment of the computerized biomedical EMMS (e-Upkaran) was carried out using a systematic review of the literature and benefits evaluation (BE) [5]. A review of the literature was done to highlight the importance of computerized medical equipment management and maintenance system and appraise the challenges and benefits associated with such systems as compared to the traditional pen-paper register. BE focused on system quality, information and service quality, use and user satisfaction, and net benefits [5]. Information was gathered based on available documents like published articles and websites. Field observation was done by visiting the concerned health facilities and data were obtained from specific hospital staff, technicians, biomedical engineers, staff involved in the maintenance, and other users of e-Upkaran. A multi-indicator stakeholder questionnaire survey was designed for this study, and each health facility was surveyed for eight selected medical equipment. The medical equipment to be included in the study was finalized after meeting with relevant stakeholders and their common availability at healthcare facilities and Mukhyamantri Nishulk Janch Yojana (MNJY) program for comparison [6]. The questionnaire focused on the effectiveness of the e-Upkaran based on the BE framework.

## Results

Review of literature

**Figure 1:**
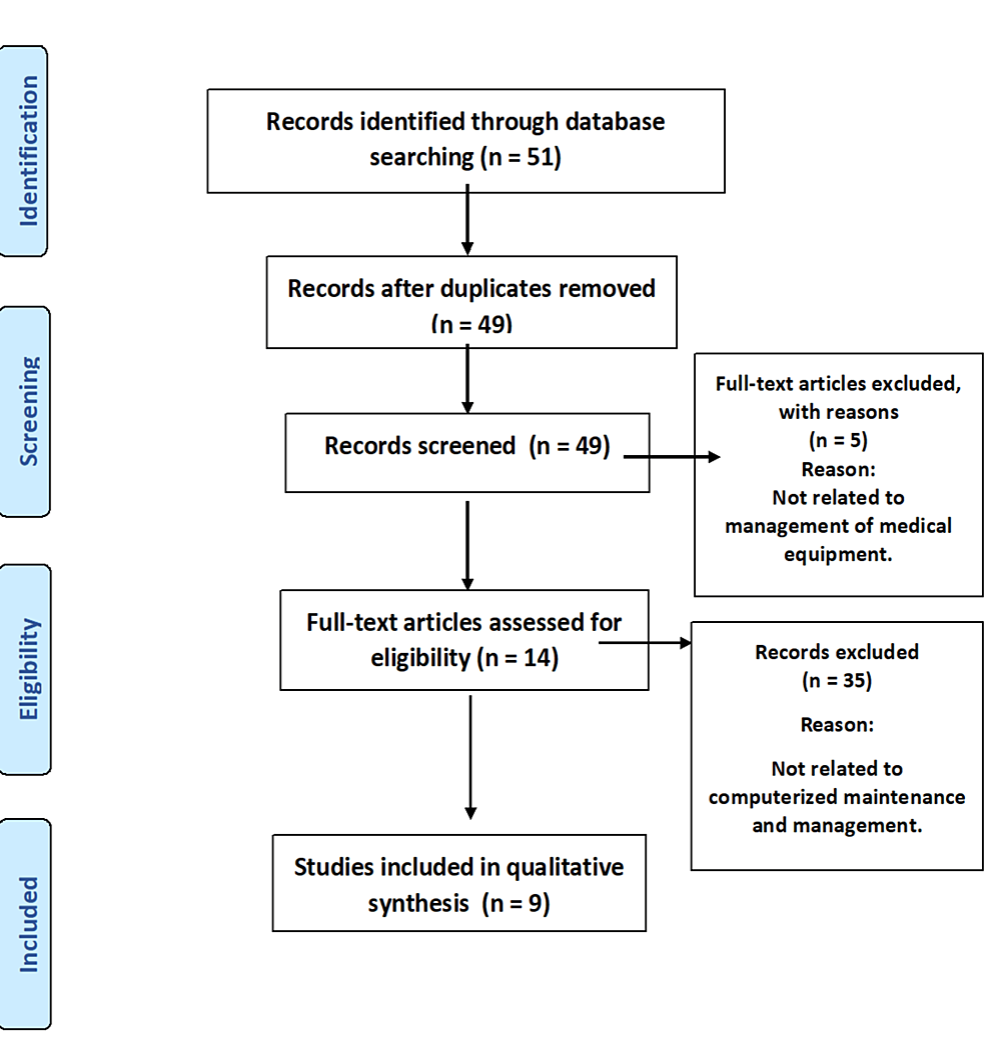
PRISMA flow diagram for the literature search process PRISMA: Preferred Reporting Items for Systematic Reviews and Meta-Analyses.

A total of 51 relevant papers published between the years 2000 and 2019 were reviewed. The Preferred Reporting Items for Systematic Reviews and Meta-Analyses (PRISMA) flow diagram for the literature search process is shown in Figure 1. Nine papers were included in the final analysis; seven studies mentioned the improvement in system quality, two studies revealed that computerized systems are better and easier to use in bigger facilities, and two studies recognized that implementation of EMMS improves the maintenance practices and work quality, reduces the maintenance cost, and promotes the safety of medical equipment. Though computerized systems are better and easier to use in health facilities, they may be troublesome for users who lack sufficient computer skills and training. The summary characteristics including the name of the study, study design, authors and year, geographic region, and importance of medical equipment maintenance and management were described (Table 1).

**Table 1:** Key findings of literature included in the review

Study design	Authors and year	Geographic region	Key findings
Not specified	Chien et al. (2010) [7]	Taipei, Taiwan	It efficiently improved the operation management of medical devices immediately and continuously.
Not specified	Mutia et al. (2012) [8]	Kenya	With help of the computer program, adequate time is created for repairing the faulty equipment thus improving the efficiency of maintenance management in hospitals.
Not specified	Shenglin et al. (2012) [9]	China	There are lots of benefits to using a computerized maintenance management system. It can provide abundant information both for clinical engineers and clinical department users, help reduce clinical use errors, improve service efficiency and increase the overall quality of clinical engineering department and hospital equipment.
Not specified	Aruna et al. (2018) [10]	Dubai, UAE	Computerized system software is the need of the hour for managing biomedical equipment. This will save many man-hours and also make the system more efficient.
Cross-sectional study	Batawalage et al. (2017) [11]	Kalutara, Sri Lanka	Equipment management processes can be re-engineered by implementing of computerized information management system for having a better quality hospital equipment management system.
Not specified	Osman et al. (2015) [12]	Sudan	Implementation of the computer program improved the maintenance practices by detecting the faults in the shortest duration. It reduced the time allocated for the repair of equipment because the information required will be accessed immediately. The facility maintenance management practices and processes and the quality of patient care will be improved. Paperwork and loss of data in maintenance management will be reduced.
Not specified	Hamdi et al. (2012) [13]	Jordan	The system proved highly efficient in minimizing equipment downtime based on healthcare delivery capacity, and, consequently, patient outcomes. The system is expected to improve the reliability of medical equipment and significantly improve safety and cost-efficiency.
Review study	Bahreini et al. (2019) [14]	Iran	Performance and safety control, activities documentation, and using computerized systems for preventive maintenance were among the issues mentioned in designing a model of medical equipment management for Iranian hospitals.
Not specified	Medenou et al. (2019) [15]	Benin, West Africa	BG Maint-KM Benin tool improved maintenance practices by making rapid failure analysis possible and allowing prioritization of medical device interventions, which was not possible on the pen-and-paper registry.

Benefit evaluation

A total of 10 health facilities were surveyed for eight selected major medical equipment to explore the state of biomedical equipment maintenance and management before and after the introduction of the e-Upkaran system.

System quality

e-Upkaran-based biomedical equipment maintenance system has the potential to improve reliability and availability (Table 2). Comparison of traditional biomedical equipment maintenance systems and e-Upkaran shows that implementation of e-Upkaran resulted in improved uptime and reduced downtime of all the study equipment. The e-Upkaran system has also improved the mean time to repair (MTTR) of equipment. After implementation of the e-Upkaran, electrocardiogram (ECG) MTTR decreased by 2.6 times and X-ray MTTR decreased by 2.5 times. For the sonography machine, no breakdown was observed in the study period, so MTTR was zero. Before the implementation of e-Upkaran, the perceived MTTR for the sonography machine was 5.7 days. A comparison of the uptime, downtime (healthcare facilities before and after having the e-Upkaran system), and response time of the selected equipment is listed in Table 3.

**Table 2:** Performance of the e-Upkaran system for selected biomedical equipment

Sr. No.	Equipment	No. of functional units	Medical equipment uptime		Medical equipment downtime		Response time (in days)		Mean time to repair = total downtime/number of breakdowns (in days)	
	Healthcare facility with		e-Upkaran	No e-Upkaran	e-Upkaran	No e-Upkaran	e-Upkaran	No e-Upkaran	e-Upkaran	No e-Upkaran
1.	ECG machine	20	85.75%	75.06%	14.25%	24.94%	1	16	7.4	19.15
2.	Blood cell	6	100%	92.88%	00%	7.12%	0	10	0	6.5
3.	Centrifuge machine	14	100%	95.89%	00%	4.11%	0	5	0	5
4.	Clinical chemistry analyzer	8	98.35%	94.52%	1.65%	5.48%	2	15	1.2	6.6
5.	Incubator	7	100%	95.90%	00%	4.10%	0	4	0	3
6.	Digital hemoglobin meter	10	100%	90.%	00%	9.59%	0	8	0	7
7.	X-Ray machine	13	87.95%	66.85%	12.05%	33.15%	1	15	7.3	17.92
8.	Sonography machine	5	100%	82.74%	00%	17.26%	0	20	0	5.72

**Table 3:** Comparative utilization coefficient before and after implementation of the e-Upkaran program

Equipment	Utilization coefficient	
Health facilities with	e-Upkaran	No e-Upkaran
ECG machine	47.16%	41.28%
Blood cell counter	100%	95.61%
Centrifuge machine	70%	67.50%
Clinical chemistry analyzer	98.35%	96.16%
Incubator	100%	95.89%
Digital hemoglobin meter	100%	96.16%
X-Ray machine	87.94%	84.38%
Sonography	100%	82.73%

Information and service quality

**Figure 2:**
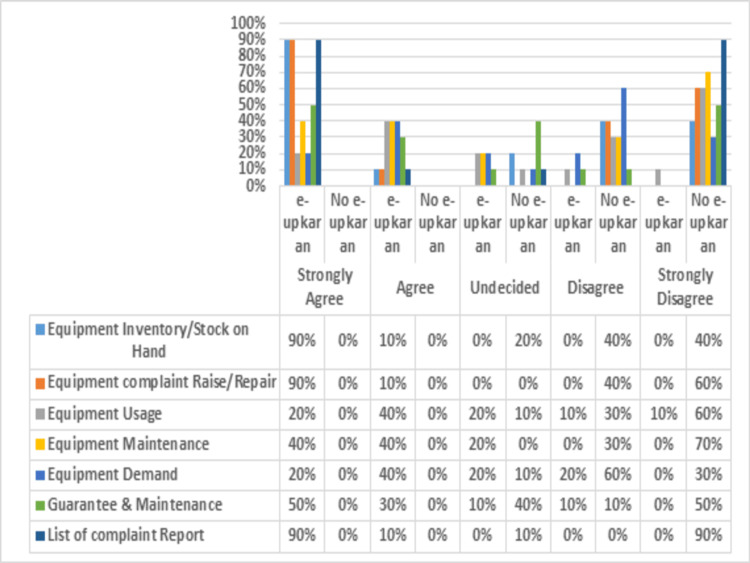
Satisfaction/agreement regarding the availability of complete and comprehensive data (reports) before and after implementation of the e-Upkaran system

A comparison of the availability of complete and comprehensive equipment inventory and management data (reports) before and after the e-Upkaran system implementation is shown in Figure 2. After the implementation of the e-Upkaran system, detailed reports of equipment inventory/stock on hand, complaints raised/repaired, and reports, use, maintenance, demand, and guarantee were readily available. The biomedical equipment maintenance service process was also simplified and lead time was reduced to two days after the implementation of e-Upkaran. Before the implementation of the e-Upkaran equipment, the service process was complex and involved a multilevel filing approval process, which required 20-25 days for responding to a complaint or requirement, resulting in a longer breakdown time for the equipment.

Use and user satisfaction

To identify the utilization of biomedical equipment, the utilization coefficient (UC) of equipment before and after the implementation of the e-Upkaran system in the health facilities was calculated. Calculation of the UC is important because optimum utilization of the biomedical equipment will result in optimal patient handling and rapid turnover, minimum possible cost, quality patient care, and patient satisfaction. Table 3 illustrates that e-Upkaran has improved the utilization of all biomedical equipment. For the e-Upkaran system, the mean satisfaction level regarding receiving easy-to-understand information was 4.5 and regarding the system user interface was 4.3 (lies between agree and strongly agree). On the contrary, it was 1.5 and 1.3 for traditional biomedical equipment systems, respectively.

Net benefit

After the implementation of the e-Upkaran system, overall equipment effectiveness (OEE) for critical equipment, namely, ECG, X-ray machine, and sonography increased to 21.94%, 15.34%, and 52.34%, respectively; hence, contributing to improved patient outcomes. The e-Upkaran system has been successful in increasing the uptime, hence reducing the risk to patients and preventing adverse health outcomes. Table 4 shows the comparison of biomedical equipment uptime and increased productivity of all equipment due to the e-Upkaran system. The cost of service ratio for the e-Upkaran system was calculated to be 1.9%. The benchmark value of the cost of service ratio is considered outstanding if it is less than 2% and good if less than 5%. Thus, the financial performance of the e-Upkaran system can be considered as outstanding. Moreover, this system has also contributed to reducing out-of-pocket expenditure (Table 5).

**Table 4:** Comparison of biomedical equipment uptime and productivity

Equipment	Uptime after implementation of the e-Upkaran system	Uptime before implementation of the e-Upkaran system	Uptime increased after the implementation of the e-Upkaran system	Increased productivity (in terms of tests performed)
ECG machine	85.75%	75.06%	10.69%	723
X-Ray machine	87.95%	66.85%	21.1%	6836
Sonography machine	100%	82.74%	17.26%	1158
Blood cell counter	100%	92.88%	7.12%	1668
Centrifuge machine	100%	95.89%	4.11%	558
Incubator	100%	95.90%	4.1%	360
Digital hemoglobin meter	100%	90%	10%	2204
Clinical chemistry analyzer	98.35%	94.25%	5.75%	759

**Table 5:** Out-of-pocket (OOP) health expenditure saved INR: Indian rupee.

Equipment	Increased productivity with the e-Upkaran system (in terms of tests performed)	The average cost of the test (in INR)	Out-of-pocket health expenditure saved (in INR)
ECG machine	723	70	50,610
X-ray machine	6836	340	2,324,240
Sonography machine	1158	740	856,920
Blood cell counter	1668	240	400,320
Clinical chemistry analyzer	1329	330	438,570
Digital hemoglobin meter	2204	60	132,240

## Discussion

This study provides a comprehensive critical overview of the effectiveness of the computerized biomedical EMMS (e-Upkaran). Implementation of e-Upkaran significantly improved uptime and reduced downtime and MTTR of equipment as compared to the traditional pen-paper system. Seven studies in the review also mentioned the improvement in system quality. According to studies conducted by Chien et al. [7], Mutia et al. [8], and Medenou et al. [15], EMMS efficiently improves the functionality and operational management of medical equipment. This system offers unique opportunities to augment medical device organization and management [10-15]. A review of the studies revealed that EMMS serves as a fundamental information resource in health care for tracking and assessing equipment performance and maintaining the accuracy of inventory records [13,16]. This study also revealed that the e-Upkaran system has enhanced service quality by simplifying the equipment maintenance service process and reducing lead time and this system seems to be a precursor of a good quality hospital equipment management system [11]. It was also evident that computerized systems are better and easier to use in bigger facilities but may be troublesome for users with inadequate computer skills and training [9,10]. A user-friendly interface could provide easy and safe access to the system [9].

The third dimension of the biomedical equipment system is the net benefit, which refers to care quality, access, and productivity [5,17]. Implementation of EMMS improves maintenance practices by detecting faults in the shortest duration, improving the quality of work, cutting maintenance costs, and ensuring the safety of medical equipment [7,8]. An effective EMMS is designed to minimize downtime and maintain equipment in good operating condition, further increasing the reliability and availability of medical equipment [12,13]. This application is also beneficial in the risk management of medical equipment [9]. A computerized system could assist in solving faults in medical equipment in hospitals, thus improving the efficiency of maintenance management in hospitals [8].

The Biomedical Equipment Management and Maintenance Program launched by the Ministry of Health and Family Welfare (MoFHW) in India provides help and support to the state governments [3]. The program is intended to “outsource medical equipment maintenance comprehensively for all facilities to improve the functionality and life of equipment.” The objective of this program is to improve healthcare management services in public health facilities, reduce the cost of care, and improve the quality of care. The aim is to “assure upkeep time for medical equipment in primary health centre/community health centre/district hospital at 85%, 90%, and 95%, respectively” [3]. Rajasthan and Gujarat have initiated this program by using a computerized web-based application that deals with the management and maintenance of equipment and instruments from one platform [17,18]. The initiative taken by these two states will be beneficial in managing and maintaining medical equipment. As India aims to strengthen health services under the Ayushman Bharat Program, with attention to both computerization and the use of information technology for better health services and referral systems, the opportunity should be used by other states as well to undertake this type of initiative for improving their biomedical equipment management and maintenance system [19,20].

## Conclusions

The e-Upkaran system has improved overall medical equipment management at the primary and secondary facility level. This system is competent in terms of improving information quality, use, and net benefit, especially for primary health centers where resources are scarce. Indian states could also adopt this system to improve their biomedical equipment management and maintenance system. Medical equipment management information data are useful for planning and monitoring the performance of medical equipment, preventive maintenance, corrective maintenance, calibration, and user training. As India aims to progress toward universal health coverage, the appropriate use of various approaches related to health information systems as well as the use of computerized systems for various purposes is needed. It will be helpful in the efficient use of available resources in government facilities without overburdening healthcare manpower.
